# “Corneal Nerves, CD11c^+^ Dendritic Cells and Their Impact on Ocular Immune Privilege”

**DOI:** 10.3389/fimmu.2021.701935

**Published:** 2021-06-18

**Authors:** Jerry Y. Niederkorn

**Affiliations:** Department of Ophthalmology, University of Texas Southwestern Medical Center, Dallas, TX, United States

**Keywords:** contrasuppressor cells, nerves, dendritic cells, immune privilege, cornea

## Abstract

The eye and the brain have limited capacities for regeneration and as such, immune-mediated inflammation can produce devastating consequences in the form of neurodegenerative diseases of the central nervous system or blindness as a result of ocular inflammatory diseases such as uveitis. Accordingly, both the eye and the brain are designed to limit immune responses and inflammation – a condition known as “immune privilege”. Immune privilege is sustained by physiological, anatomical, and regulatory processes that conspire to restrict both adaptive and innate immune responses.

## Introduction

**Figure 1:**
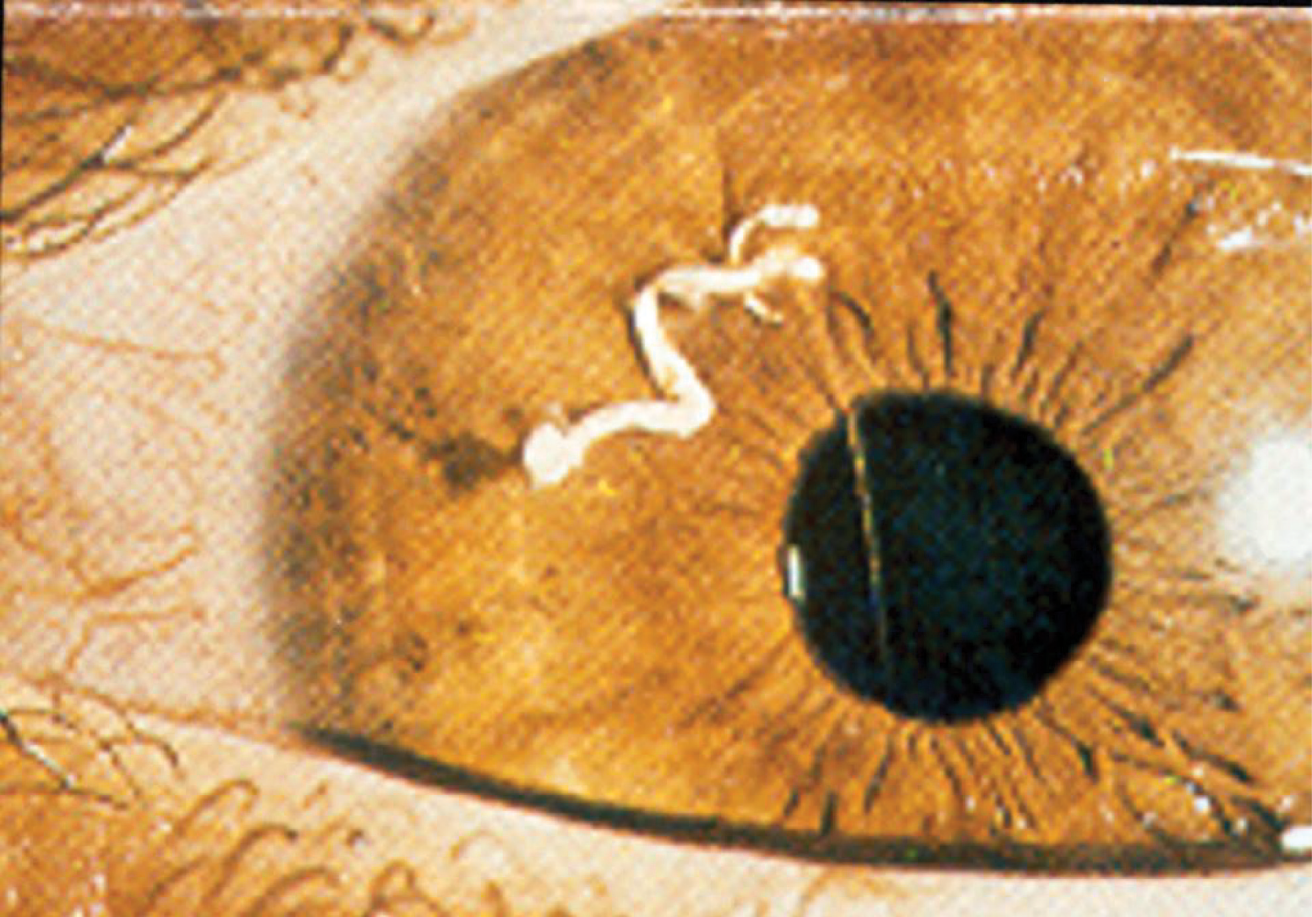
Germinating seedling in the anterior chamber of an eight year-old boy following 10 weeks of ocular pain. The vermiform object was extirpated and identified by a botanist to be a seedling of a dicotyledonous plant. Reproduced from Abel (1) with permission from Archives Ophthalmology.

A striking example of immune privilege was reported by Abel who treated an 8-year old boy who had a 10 week history of ocular pain (1). Examination of the painful eye revealed the presence of a vermiform foreign body in the anterior chamber. The foreign body was removed and it was found not to be a helminth but rather, it was a seedling of a dicotyledonous plant of the Compositae family that had germinated in the anterior chamber (**Figure 1**). Two noteworthy features of this case bear noting. The “foreign body” had eluded the immune system and persisted in the eye for over two months. The second and equally astonishing finding was the conspicuous absence of inflammation in the anterior chamber of the eye in which the seedling had germinated. These two observations are in keeping with the principles of immune privilege: 1) the prolonged survival of a foreign tissue that would normally be rejected in conventional body sites and 2) the local quenching of inflammation. A more commonly recognized example of ocular immune privilege is the remarkable success of corneal allografts, which in uncomplicated cases enjoy a survival rate of 90% even though histocompatibility matching is not routinely practiced and the only immunosuppressive drugs are topically applied corticosteroids (2–4).

## Immune Privilege Is a Dynamic Process That Involves Anatomical, Physiological, and Immunoregulatory Elements

Although the concept of immune privilege is widely recognized it is often over-simplified and misunderstood. Two common misconceptions about immune privilege are that corneal transplants are exempt from immune rejection and that the anterior chamber (AC) of the eye is devoid of lymphatic drainage, which presumably isolates the interior of the eye from the systemic immune apparatus. Although corneal transplants enjoy a survival that is unrivaled in the field of transplantation, they can occasionally undergo immune rejection, which remains the leading cause of corneal graft failure. The notion that the AC of the eye is devoid of lymphatic drainage was proposed by Medawar over a half-century ago (5) and persisted until the late 20^th^ century when it was discovered that although lymphatic vessels were invisible to most anatomical observations, antigens introduced into the AC could reach the ipsilateral cervical lymph nodes (6), however the majority of antigens injected into the AC enter the venous circulation and reach the lymph nodes *via* the blood vascular pathway (7).

Over the past 50 years it has become clear that immune privilege in the AC and the success of corneal allografts are due to a combination of anatomical, physiological, and dynamic immunoregulatory processes that restrict the expression of both innate and adaptive immune responses (8, 9). In addition, many of the blood vessels within the eye are non-fenestrated and the tight junctions between the cells lining the blood vessels restrict the diapedesis of leukocytes and the extravasation of macromolecules into the interior of the eye (10). This “blood-ocular barrier” is imperfect and in certain conditions leukocytes can extravasate and enter the eye, a condition that occurs in uveitis.

The aqueous humor that fills the AC of the eye is a potpourri of anti-inflammatory and immunosuppressive molecules including: a) transforming growth factor-β(TGF-β), b) α-melanocyte stimulating hormone (α-MSH), c) complement regulatory proteins (CRP), d) calcitonin gene-related protein (CGRP), e) somatostatin (SOM), f) macrophage migration inhibitory factor (MIF), and g) vasoactive intestinal peptide (VIP) (11–16). These humoral molecules act to quench inflammation and to buffer the deleterious effects of activation of the complement cascade. The cells lining the inside of the eye are decorated with a panoply of cell membrane-bound molecules that induce apoptosis of immune cells that enter the eye thereby eliminating immune attack. These include: a) FasL, b) tumor necrosis factor-related apoptosis-inducing ligand (TRAIL), c) PD-L1, d) CRP, and e) galectin-9 (17–19).

In addition to the anatomical and physiological properties of the eye, dynamic immunoregulatory processes contribute to ocular immune privilege. Antigens entering the eye including histocompatibility antigens sloughed from the corneal endothelium of corneal transplants induce a dynamic form of immune deviation in which cell-mediated immune responses such as delayed-type hypersensitive (DTH) and cytotoxic T lymphocyte (CTL) responses are suppressed by T regulatory cells (Tregs) – a phenomenon termed anterior chamber-associated immune deviation (ACAID) (8). Not only does ACAID suppress Th1 type immune responses but it also blunts Th2-mediated inflammatory disease (20). ACAID also culminates in a deviation of the antibody responses from complement fixing isotypes to a preferential production of non-complement-fixing antibodies. This deviation reduces the likelihood of antibody-mediated injury to ocular tissues as activation of the complement cascade can produce deleterious effects of granulocytic inflammation that occurs in response to complement byproducts.

## Role of Antigen-Presenting Cells in Corneal Allograft Survival and ACAID

**Figure 2:**
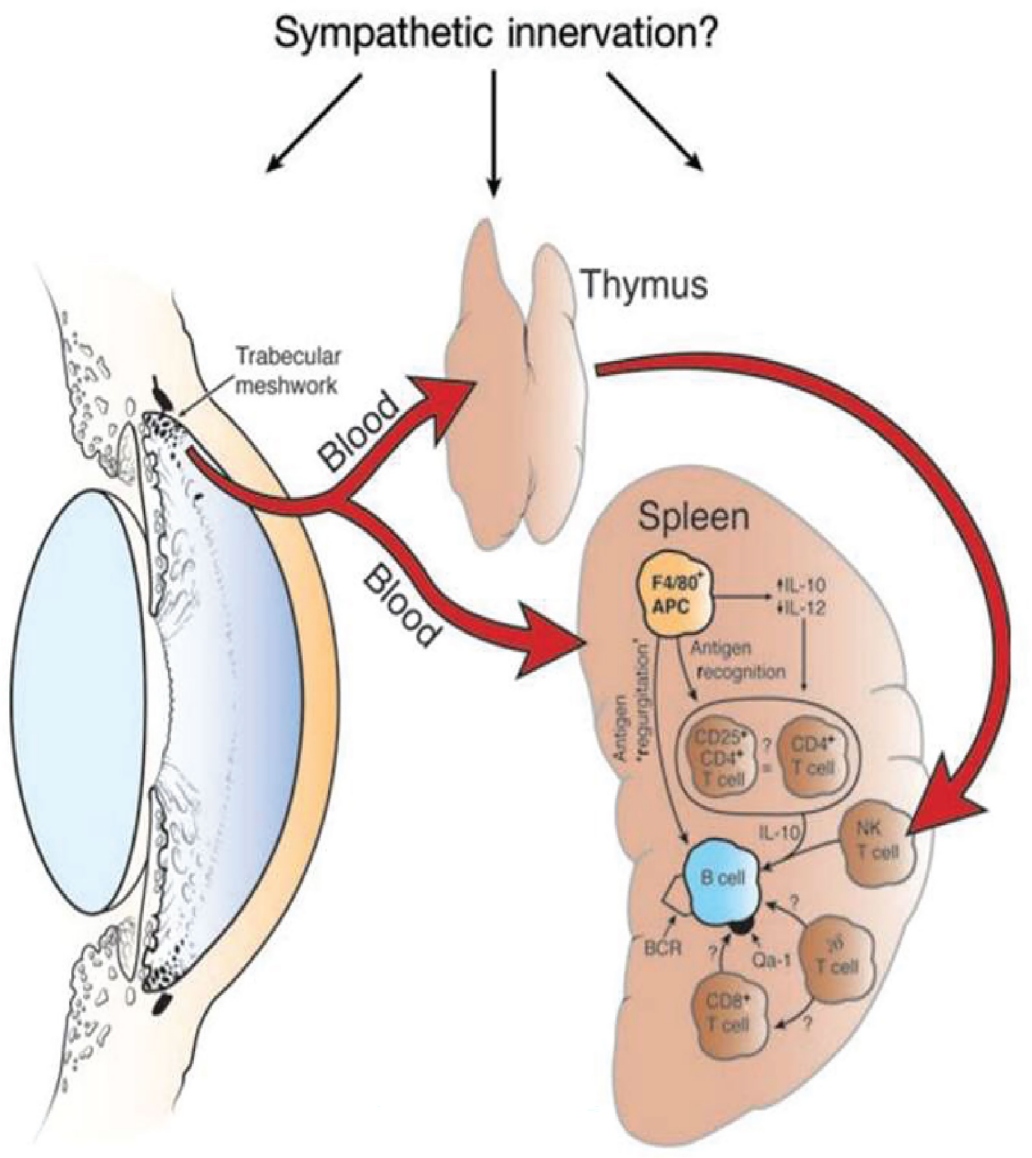
Four organ systems are required for the induction of ACAID. Removal of the eye, spleen, or thymus within 3 days of injecting antigen into the anterior chamber prevents the induction of ACAIDS. Chemical sympathectomy prior to injecting antigens into the anterior chamber also prevents ACAID. BCR, B cell receptor. Reproduced from Niederkorn (9) with permission from Nature Publishing Group.

The induction of ACAID is an extraordinarily complex process that involves four organ systems: a) eye, b) spleen, c) thymus, and d) sympathetic nervous system. Removal of the eye, thymus, or spleen within three days of introducing antigens into the AC abrogates the induction of ACAID. Likewise chemical sympathectomy prior to AC injection of antigens blocks the induction of ACAID. The contributions of these organ systems in the induction of ACAID are complex and are discussed in detail elsewhere (8) (**Figure 2**).

The eye-dependent stage of ACAID is widely believed to involve the participation of antigen-presenting cells (APC), which under the influence of soluble molecules in the AC, induce the generation of Tregs. Antigens are captured by resident F4/80^+^ APC that are bathed in TGF-β, which in turn preferentially produce IL-10. The production of IL-10 by ocular APC is pivotal to induction of ACAID as shown by the inability of ocular APC from IL-10 knockout mice to induce ACAID (21). Ocular APC exposed to TGF-β that is present in the AC preferentially produce macrophage inflammatory protein-2 (MIP-2), which is crucial for the induction of T regs in the spleen (22). The role of F4/80^+^ APC in the induction of ACAID is strengthened by the observation that ACAID cannot be induced in F4/80 KO mice (23).

ACAID is intimately associated with corneal allograft survival (9, 24) Maneuvers that block the induction of ACAID, such as splenectomy or deletion of IL-10, invariably result in the immune rejection of corneal allografts. Likewise, induction of ACAID through the intracameral injection of corneal graft donor alloantigens prior to corneal transplantation significantly enhances corneal allograft survival and dramatically reduces the incidence of immune rejection (25).

## Role of CD11c^+^ DC in the Abrogation of ACAID and Induction of Corneal Allograft Rejection

The remarkable success of corneal allografts defies the laws of transplantation immunology. Studies in animal models of penetrating keratoplasty demonstrate that in the absence of any form of immunosuppressive agents corneal allografts mismatched with the recipient at all known MHC and minor histocompatibility gene loci routinely enjoy a 50% acceptance rate (4). By contrast orthotopic skin allografts transplanted under similar conditions undergo rejection in 100% of the hosts. As mentioned earlier, immune privilege is not absolute and corneal allografts can undergo immune rejection. Virtually any condition that provokes inflammation at the ocular surface, such as herpes simplex virus keratitis (HSVK), abolishes ocular immune privilege and invariably leads to corneal allograft rejection (4, 26).

The leading risk factor for corneal allograft failure in human subjects is the rejection of a previous graft. The incidence of corneal allograft rejection rises three-fold in patients receiving a second corneal transplant (27). On first blush one might reasonably conclude that such patients were sensitized to the donor alloantigens expressed on the first transplant and the heightened incidence of rejection was simply a recall or memory immune response elicited by the second transplant. However, this conclusion is flawed. Under normal conditions corneal buttons are selected for transplantation based on the quality of the tissue and the corneas suitability for surgery. Histocompatibility matching is not employed and accordingly the array of MHC and minor histocompatibility antigens expressed on the first and second grafts is random and the likelihood of shared antigenic epitopes on the first and second grafts is remote. Moreover, second corneal allografts can occur in patients with a long-standing clear transplant in the opposite eye suggesting that the immune system was “unaware” of the alloantigens expressed on the first transplant (28).

**Figure 3:**
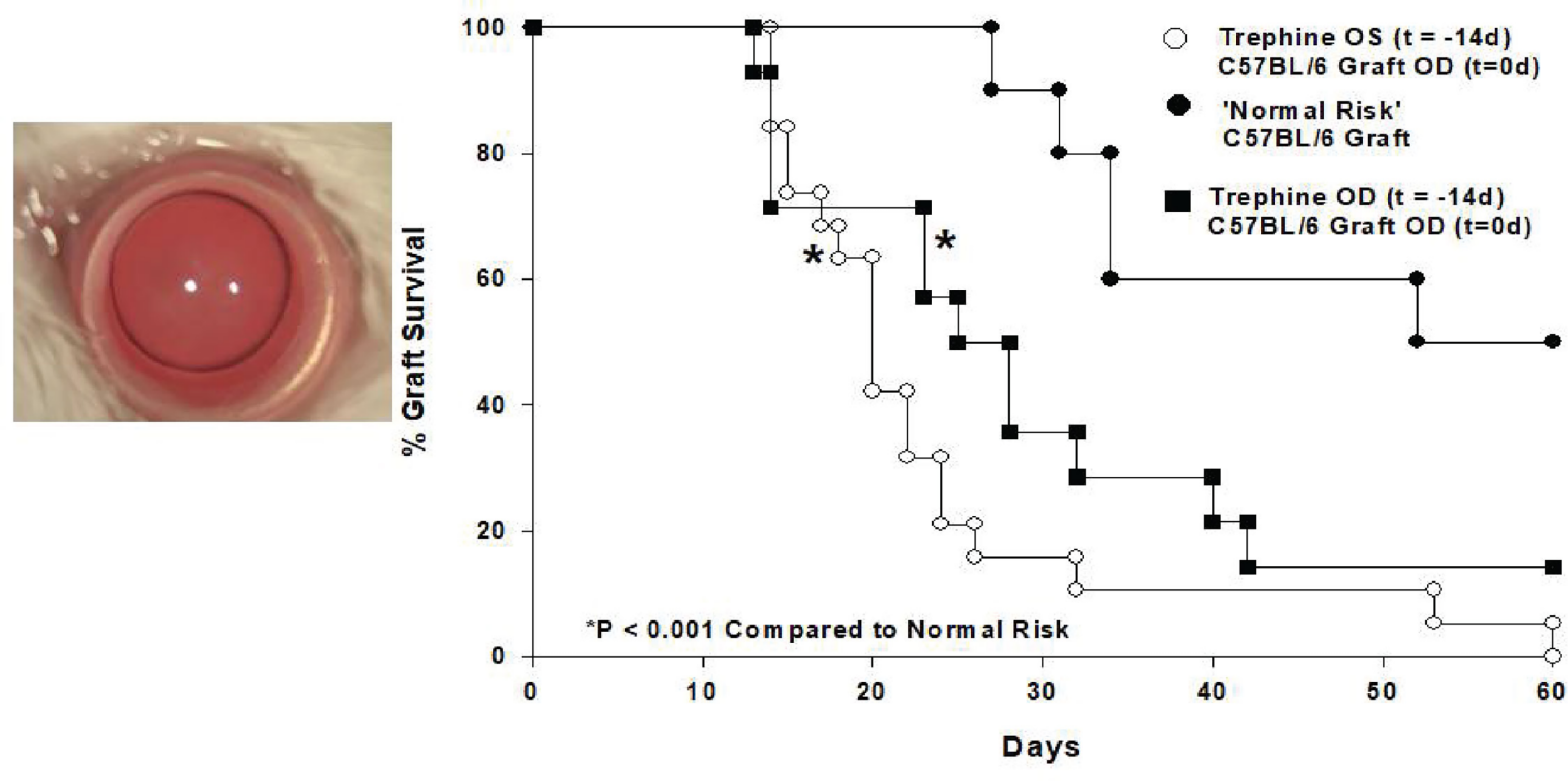
Shallow circular incisions of the cornea in one eye abolishes ACAID in both eyes. 360° corneal incisions were placed in left (OS) eyes of BALB/c mice 14 days prior to application of C57BL/6 corneal allografts to the right (OD) eyes. (Modified from Paunicka et al. (29).

In an attempt to unravel this paradox we employed a mouse model of penetrating keratoplasty and recapitulated experiments in which patients receive two corneal transplants from unrelated donors. For these experiments we employed a widely utilized mouse model in which C57BL/6 corneal allografts are transplanted to BALB/c recipients. These two mouse strains differ at all known major and minor histocompatibility gene loci, yet only 50% of the corneal allografts undergo immune rejection even though no immunosuppressive drugs are employed. To evaluate the effect of a first corneal transplant on the survival of subsequent corneal grafts we transplanted corneas from C3H mice to the right eyes of BALB/c recipients. Sixty days later we transplanted C57BL/6 corneas to the opposite eye (i.e., left eye) of these mice. Instead of the expected 50% incidence of rejection for initial C57BL/6 corneal allografts in naïve hosts, we observed 100% graft rejection (29). Importantly C3H and C57BL/6 mice differ at all known MHC and minor histocompatibility gene loci and thus the C3H corneal allografts were incapable of immunizing the BALB/c recipients to C57BL/6 alloantigens thereby removing the possibility that the profound increased incidence of rejection in the BALB/c mice was a result of “cross-immunization” or prior allosensitization. Thus, corneal surgery itself robbed the second eye of its immune privilege. To confirm that the surgery and not the rejection of a previous corneal graft abolished immune privilege, syngeneic BALB/c corneal grafts were placed onto the right eyes of BALB/c mice 60 days prior to transplanting C57BL/6 corneal allograft to the opposite eyes. As expected, the BALB/c syngrafts remained clear and healthy throughout the entire course of these experiments, yet 100% of the C57BL/6 corneal allografts applied to the opposite eye underwent immune rejection even though the expected incidence of rejection was 50%. These results recapitulated the previously reported cases in human subjects in which second corneal transplants underwent rejection in hosts having long-standing clear first corneal transplants in the opposite eye (28). Additional experiments revealed that this loss of immune privilege was due to the severing of corneal nerves that occurs during the penetrating keratoplasty procedure. Simply cutting the superficial corneal nerves with a circular trephine in one eye abolished immune privilege in the opposite eye and thus represented a “sympathetic loss of immune privilege” (SLIP) (29). SLIP did not require complex surgical procedures and could be induced by simply making a shallow circular incision in the corneal epithelium (**Figure 3**).

It is important to note that SLIP should not be confused with a similar condition called sympathetic ophthalmia (SO) that sometimes occurs in individuals following a penetrating traumatic injury to one eye that is subsequently followed by intense inflammation in the opposite eye (30). The inflammation in the “sympathizing” eye led to the moniker sympathetic ophthalmia. Although SO is still poorly understood, it is widely believed that the penetrating injury to one eye leads to the release of ocular antigens, especially those sequestered from the immune apparatus in the retina, and culminates in the generation of an immune response to the ocular antigens and the appearance of immune inflammation in both eyes. By contrast, SLIP is not the direct initiation of an antigen-specific immune response, instead it is the antigen non-specific disabling of Tregs and can be induced by a single bolus injection of 0.1 pg of substance P (SP) and thus, occurs in the absence of traumatic injury to eye (see below).

The impact of neuropeptides on immune responses has long been recognized. Neuropeptides exert an important effect on immune privilege in the eye (11). Interestingly, one neuropeptide stands apart from the rest. The expression of SP and its receptor NK1-R increases over 300% in both eyes following circular corneal incisions in one eye (29). Blocking NK1-R with the SP receptor antagonist Spantide II, restores immune privilege in mice subjected to corneal nerve injury and results in 50% corneal allograft acceptance, which is identical to the incidence of graft survival in naïve mice. Moreover, simply injecting as little as 0.1 pg of SP intravenously abrogates immune ocular immune privilege and results in 100% corneal allograft rejection.

Severing corneal nerves also adversely affects ACAID. Placing a shallow corneal incision in one eye prevents the induction of ACAID in both eyes (31, 32). These results are remarkably similar to earlier finding by Lucas and co-workers who observed that retinal laser burns (RLB) to one eye prevented the induction of ACAID in the opposite eye that was not subjected to RLB (33). Moreover, the loss of ACAID produced by RLB coincided with a steep upregulation in the SP receptor NK1-R in the opposite non-manipulated eye. Studies by Guzman and co-workers found a similar association between corneal nerve injury and the induction of ocular immune tolerance (34). These investigators found that placing 180° incisions in the cornea of one eye prevented the induction of mucosal tolerance following topical application of ovalbumin (OVA) to the opposite eye. This abrogation of mucosal tolerance, like SLIP, could be reversed by the application of a SP receptor antagonist.

SP has emerged as the end stage effector molecule in SLIP. Although the SP receptor, NK1-R is expressed on numerous cell types including antigen-presenting DCs, we were intrigued by the juxtaposition of CD11c^+^ ocular surface DCs to the area where we placed circular incisions in the cornea. When stimulated *via* their NK1-R, CD11c^+^ DCs inhibit IL-10 production and promote the generation of Th1 immune responses (35). Both of these conditions are associated with the abrogation of ACAID and corneal allograft rejection (4, 9).

We explored the potential role of CD11c^+^ DCs in the development of SLIP and the loss of ocular immune privilege by isolating CD11c^+^ CS from mice subjected to corneal nerve injury (i.e., trephining) and adoptively transferring them to naïve recipients. ACAID could not be induced in these recipients but was induced in recipients of CD11c^+^ DCs from donors that had not been subjected to corneal nerve injury (31). Additional experiments revealed that CD11c^+^ DCs isolated from trephined donors blocked the suppressive activity of Tregs *in vivo*. Accordingly, we categorized these CD11c^+^ cells as contrasuppressor (CS) cells based on their capacity to suppress Tregs and their similarity to “contrasuppressor” cells described over four decades ago (36). Although the concept of “suppressor cells” was hotly debated for over a decade, the suppressor cell concept vindicated by the elegant investigations of Sakaguchi who now referred to these cells by the euphemistic moniker “T regulatory cells” (37). CS cells have also enjoyed a renewed appreciation and have been demonstrated in multiple models of immune regulation (38).

## Induction of CD11c^+^ CS Cells and Their Mode of Action

**Figure 4:**
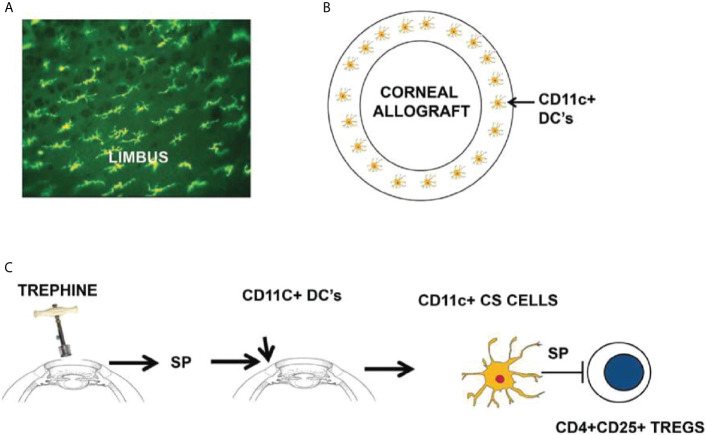
Sympathetic loss of immune privilege is the result of an axis involving ocular surface CD11c^+^ DCs, corneal nerves, and the elaboration of substance P (SP). **(A, B)** MHC class II^+^ DCs (including CD11c^+^ DCs) reside at the interface between the corneal allograft and the graft bed. **(C)** Severing corneal nerves with a trephine elicits the release of SP that converts resident CD11c^+^ DCs to contrasuppressor (CS) cells that disable CD4^+^CD25^+^ Tregs. Reproduced from Niederkorn (39) with permission from Invest Ophthalmol Vis Sci.

The weight of evidence suggests that there is an axis involving ocular surface CD11c^+^ cells, corneal nerve injury, and the elaboration of SP that conspires to rob both eyes of their immune privilege (**Figure 4**). SP is clearly involved in the generation of CS cells as *in vitro* treatment of CD11c^+^ cells with SP licenses them to block Treg activity *in vivo* (31). Moreover, corneal nerve injury fails to induce CS cells in SP ^-/-^ mice (31). A single bolus intravenous injection of 1.0 pg prevents the induction of ACAID and 0.1 pg of SP culminates in 100% corneal allograft rejection (29, 31). The pivotal role of SP in the generation of CS cells was confirmed by briefly exposing naïve CD11c^+^ DC to SP *in vitro* and infusing these cells into naïve recipients. Mice that received SP-conditioned CD11c^+^ DC resisted the development of ACAID, while neither naïve CD11c^+^ DC nor CD11c^+^ cells conditioned in saline adversely affected the generation of ACAID T regs (32). We are attracted to the hypothesis that SP elaborated in response to corneal nerve injury converts resident CD11c^+^ DC at the graft/host margin to become CS cells. If this scenario is correct then it should be possible to prevent the generation of CS cells by purging ocular CD11c^+^ DCs prior to corneal nerve injury. This hypothesis was confirmed in experiments in which clodronate-containing liposomes were injected into the conjunctiva prior to trephining the ocular surface (32). Subconjunctival injection of clodronate-containing liposomes specifically depletes ocular surface APC including CD11c^+^ DC (40). Corneal nerve injury blocks ACAID, however depletion of ocular surface CD1c^+^ DC through the injection of clodronate-containing liposomes prior to AC injection of antigens restores ACAID and allows the development of Tregs (32). The crucial role of SP signaling in the generation of CD11c^+^ CS cells was also supported by experiments in which administration of the SP receptor antagonist Spantide II prevented the generation of CS cells and restored immune privilege of corneal allografts in hosts subjected to trephining and simultaneously injected with Spantide II (29, 31).

CS cells disable two distinct categories of T regulatory cells that are involved in ocular immune privilege: a) CD8^+^ ACAID T regs and b) CD4^+^CD25^+^ corneal allograft-induced Tregs. In both cases the CS cells exert their effect in an antigen-non-specific manner. The molecular mechanisms involved in Treg suppression are still under investigation, but at least one molecule has emerged as a crucial player. Microarray analysis on 12,000 genes expressed on CD8^+^ ACAID T regs revealed an 85-fold increase in the expression of CD103 (41). The finding that ACAID cannot be induced in CD103^-/-^ mice supports the notion that CD103 is critical for the induction of CD8^+^ ACAID T regs (41). It bears noting that CD103 is also expressed on CD4^+^CD25^+^ T regs (41–45). With this in mind we examined the role of CD103 in CS cell blockade of Treg activity. Co-culturing CD8^+^ ACAID T regs with CS cells resulted in a 66% reduction in CD103 expression on ACAID T regs (46). The down-regulation of CD103 on CD8^+^ ACAID Tregs was directly attributed to SP and could be mimicked by co-culturing CD8^+^ T regs with SP *in vitro* (46). CD103 is a cell-adhesion molecule that binds to E-cadherin, which is expressed on numerous cell types including APCs (47, 48). TGF-β is necessary for conditioning ocular APC for the induction of ACAID presumably by upregulating E-cadherin. CD103/E-cadherin interactions are enhanced when T cell-receptor signaling occurs (49). These interactions would stabilize the interaction between CD8^+^ ACAID Tregs and effector T cells that occurs during the suppression of T cell responses and it would also explain the requirement for CD103 expression on CD8^+^ ACAID Tregs for their capacity to directly suppress T cells during DTH responses.

## Conundrums and Puzzling Properties of SLIP

On the surface, the induction of SLIP and the generation of CS cells seem straightforward and logical. However, closer analysis reveals conundrums that are difficult to reconcile. A single intravenous bolus injection of as little as 0.1 pg of SP abolishes the immune privilege of corneal allografts (29). This loss of immune privilege persists for at least 100 days even though only a single injection of SP was administered and the serum half-life of SP is less than two minutes (50). The nature of the corneal nerve injury involved in the development of SLIP is also puzzling. Circular corneal incisions induce CD11c^+^ CS cells yet “X” shaped corneal incisions of similar linear dimensions do not adversely affect corneal allograft survival or ocular immune privilege (29). Equally perplexing is the observation that blocking the SP receptor with Spantide II prevents SLIP but does not enhance corneal allograft survival beyond the 50% acceptance that is found in naïve mice. That is, blocking SP restores immune privilege, but does not enhance it. DC are recognized for their remarkable capacity to amplify immune responses and as few as 10 allogeneic DC can elicit allospecific T cell responses (51) and as little as 1,000 CD11c^+^ CS cells can abolish immune privilege and ablate the suppressive activity of ACAID Tregs (32).

Corneal nerve ablation or a single intravenous injection of SP abolishes immune privilege for corneal allografts for at least 100 days, which suggests that CD11c^+^ CS cell populations are long-lived. However, whole body ionizing irradiation 14 days after corneal nerve injury abolishes CS cell activity, which suggests that the initial CS cell population is radiosensitive (32). Studies in bone marrow (BM) chimeric mice revealed that lethally irradiated CD45.2^+^ congenic mice that were reconstituted with BM from CD45.1^+^ congenic donors failed to display CS cell activity in either CD45.1^+^ or CD45.2^+^ cells populations following corneal nerve injury. This finding suggests that naïve BM-derived cells introduced into mice 14 days after corneal nerve injury are not converted to CS cells. This also indicates that the generation of CS cells is complete within 14 days or corneal nerve injury and that CS cells themselves are not long-lived even though SLIP persists for at least 100 days following trephining of the cornea (32).

## What is the Meaning of SLIP?

On first blush SLIP seems to represent a flaw in ocular immune privilege. The time-honored view of ocular immune privilege proposes that the eye is endowed with a highly redundant system that dampens immune-mediated inflammation in order to preserve ocular tissues that have little or no regenerative capacities. Why then does the simple severing of corneal nerves abolish ocular immune privilege and why are both eyes affected? We favor the hypothesis that under certain circumstances terminating immune privilege is an adaptation to restore immune defense mechanisms at the ocular surface to protect the host against life-threatening infections. Under normal circumstances nominal non-infectious agents that pose no threat to survival are either ignored or they induce immune tolerance that reduces the risk of unwittingly provoking inflammation that might damage ocular tissues. By contrast, infectious agents that are potentially lethal transmit “danger signals” that lead to the termination of immune privilege. In mainstream immunology “danger signals” are in the form of pathogen-associated molecular patterns (PAMP that are recognized by toll-like receptors (TLRs) and other pathogen recognition receptors (PRRs). Although TLRs are expressed on cells comprising the ocular surface, other danger signals also originate at the ocular surface. Corneal nerve injury, alkali burns, and infectious agents can terminate immune privilege by a process that involves the release of SP, which represents another “danger signal” (39). It is noteworthy that two of the most common causes of infectious blindness, *Pseudomonas aeruginosa* and herpes simplex virus (HSV) are closely associated with the release of SP and the termination of ocular immune privilege (52–55). It is reasonable to assume that a corneal infection in one eye has a high likelihood to also occur in the opposite eye. As a result, the immune system anticipates that both eyes are at risk for life-threatening infections. A similar condition has been reported with cutaneous infections with *Candida* and *Staphylococcus* (56). Pain signals transmitted by neuropeptides during cutaneous infections with either of these microorganisms activates TRPV1^+^ sensory nerves and activates innate Th17 responses in affected tissues and in adjacent non-involved tissues in anticipation of the infections spreading. The authors of this study use the term “anticipatory immunity”, which is conceptually similar to SLIP. However, unlike “anticipatory immunity” in the skin, terminating immune privilege in the eye imposes more serious consequences. Even though life is preserved, the cost can be blindness.

## Author Contributions

The author confirms being the sole contributor of this work and has approved it for publication.

## Funding

Supported in part by P30-EY030413 and an unrestricted grant from Research to Prevent Blindness.

## Conflict of Interest

The author declares that the research was conducted in the absence of any commercial or financial relationships that could be construed as a potential conflict of interest.
